# Adipokines, cell polarity disruption and breast cancer

**DOI:** 10.18632/aging.203621

**Published:** 2021-10-06

**Authors:** Danila Coradini

**Affiliations:** 1 Department of Clinical Sciences and Community Health, University of Milan, Milan, Italy

**Keywords:** breast cancer, adipokines, adipokine receptors, cell polarity

The probability of developing invasive breast cancer increases progressively with age, but it raises sharply in women aged 45-55 years, concomitantly with the stop of the ovarian activity and the fall in the production of sex steroids hormones, especially estrogens. Apparently, in contrast with the decline of circulating estrogens, over 70% of primary breast cancer in postmenopausal patients express estrogen receptor (ER) and proliferate in response to the stimulatory effect of estrogens produced in extra-ovary districts. These extragonadal sites include the adrenal gland, which produces androgen precursors such as testosterone and androstenedione, and the adipose tissue, where the aromatase enzyme converts androgen precursors to bioactive estrogens.

Compared with the circulating estrogens produced by the ovaries, those synthesized in the adipose tissue reach a local concentration high enough to exert a significant paracrine activity on neighboring tissues. Considering that, in the mammary gland, the fat tissue may represent up to 80% of the entire volume, it is not surprising that the estrogens, locally produced, provide the suitable microenvironment for the initiation and progression of ER-positive breast cancer.

For a long considered a simple fat depot, adipose tissue is now emerging as an active tissue. Indeed, in addition to the production of estrogens by the aromatization of androgenic precursors, adipocytes produce specific growth factors, termed adipokines, which can influence neighboring cells’ behavior. For example, in the breast, leptin, and adiponectin, the two main adipokines synthesized and released by mature adipocytes, act in a tightly balanced manner during mammary gland remodeling [1]. However, cumulating evidence indicates that leptin is also involved in mammary carcinogenesis [2]. *In vitro*/*in vivo* studies demonstrated that alterations in the tissue level of leptin can disrupt the correct structural and functional organization of normal epithelial cells, the so-called cell polarity, leading to tumor initiation [3]. Besides, more than 80% of primary tumors produce leptin [4] which can affect the growth of neighboring cells by activating signaling pathways that, in turn, induce the expression of the aromatase gene, thereby contributing to the production of bioactive estrogens through the aromatization of androgens.

Cells polarity is an essential property required for the correct alignment of cells during epithelial tissue organization and consists of the asymmetric distribution of intracellular components along the apical-basal axis. Under the control of three molecular apparatus dynamically interplaying, intracellular components compartmentalize to define an apical domain, directly in contact with the luminal space, and a basal–lateral domain in contact with adjacent cells and basement membrane. Of the three molecular apparatus governing cell polarity, the domain-identity machinery plays a crucial role in establishing the so-called apical junctional complex, which includes tight and adherens junctions, and whose correct assembly requires the interplay of three specific evolutionarily conserved multiprotein structures, termed PAR, CRB, and SCRIB polarity complexes. PAR and CRB localize at the apical domain, whereas SCRIB localizes at the basal–lateral domain.

Given the complexity of the process that regulates cell polarity establishment and maintenance, it is not surprising that disruption of the interaction among the three polarity complexes can lead to apical-basal polarity lost, alteration in the structural and functional compartmentalization of intracellular components, misregulation of tissue organization, and cancer initiation [5].

A recent study [6], aimed at exploring the relationship among the expression of the genes coding for leptin and adiponectin, their receptors, and a panel of proteins involved in cell polarity, in normal and cancerous mammary tissue, showed that: 1. in normal tissue from breast reduction mammoplasty, the expression of the genes coding for adipokines receptors associated inversely with cell polarity establishment, apical junctional complex formation, epithelial tissue organization, and expression of *GGT1*, the gene encoding γ-glutamyl transferase enzyme, a marker of cell distress and membrane disruption; 2. this negative correlation progressively decreased in histologically normal and cancerous tissue.

Of particular interest was the dramatic decrease of the correlation between *ADIPOR2,* one of the genes coding for the adiponectin receptor expressed by epithelial cells, and all genes involved in cell polarity and apical junction complex establishment. Already found in the tissue surrounding the tumor and histologically classified as normal, this loss of correlation suggests that the downregulation of *ADIPOR2* could represent an early step in the process leading to a decreased uptake of adiponectin, an unbalanced leptin stimulatory activity, and cell polarity disruption.

This finding is of great clinical relevance considering the role played by weight gain/obesity in breast cancer etiology. Postmenopause is frequently associated with weight gain, mainly due to the accumulation of adipose tissue promoted by testosterone no longer balanced by ovarian estrogens [7]. The increase in adipose tissue and, in particular, the augment of fat in the abdomen (visceral or central obesity) promotes the onset of insulin resistance, which results in chronic hyperinsulinemia that, in turn, stimulates adipocytes to produce and release leptin continuously [8]. In the breast, such an unceasing insulin-dependent production of leptin by mature adipocytes, not more counteracted by the inhibitory activity of adiponectin because of *ADIPOR2* downregulation, can disrupt the correct structural and functional organization in the normal epithelial tissue and trigger tumor initiation, which is promoted and sustained by the bioactive estrogens produced by aromatization of androgenic precursors.
